# Long‐term outcome of cerebral amyloid angiopathy‐related hemorrhage

**DOI:** 10.1111/cns.13922

**Published:** 2022-08-16

**Authors:** Ruiwen Che, Mengke Zhang, Hailiang Sun, Jin Ma, Wenbo Hu, Xin Liu, Xunming Ji

**Affiliations:** ^1^ Department of Neurology Xuanwu Hospital, Capital Medical University Beijing China; ^2^ Beijing Key Laboratory of Hypoxia Conditioning Translational Medicine Beijing China; ^3^ Department of Neurosurgery Beijing Fengtai You'anmen Hospital Beijing China; ^4^ Department of Radiology Beijing Chaoyang Hospital, Capital Medical University Beijing China; ^5^ Department of Neurosurgery Xuan Wu Hospital, Capital Medical University Beijing China; ^6^ Beijing Institute of Brain Disorders Beijing China; ^7^ Capital Medical University Beijing China

**Keywords:** cerebral amyloid angiopathy, cerebral small vessel diseases, intracranial hemorrhages, neuroimaging, survival analysis, treatment outcome

## Abstract

**Object:**

The long‐term functional outcome of cerebral amyloid angiopathy‐related hemorrhage (CAAH) patients is unclear. We sought to assess the long‐term functional outcome of CAAH and determine the prognostic factors associated with unfavorable outcomes.

**Methods:**

We enrolled consecutive CAAH patients from 2014 to 2020 in this observational study. Baseline characteristics and clinical outcomes were presented. Multivariable logistic regression analysis was performed to identify the prognostic factors associated with long‐term outcome.

**Results:**

Among the 141 CAAH patients, 76 (53.9%) achieved favorable outcomes and 28 (19.9%) of them died at 1‐year follow‐up. For the longer‐term follow‐up with a median observation time of 19.0 (interquartile range, 12.0–26.5) months, 71 (50.4%) patients obtained favorable outcomes while 33 (23.4%) died. GCS on admission (OR, 0.109; 95% CI, 0.021–0.556; *p* = 0.008), recurrence of ICH (OR, 2923.687; 95% CI, 6.282–1360730.14; *p* = 0.011), WML grade 3–4 (OR, 31.007; 95% CI, 1.041–923.573; *p* = 0.047), severe central atrophy (OR, 4220.303; 95% CI, 9.135–1949674.84; *p* = 0.008) assessed by CT was identified as independent predictors for long‐term outcome.

**Interpretation:**

Nearly 50% of CAAH patients achieved favorable outcomes at long‐term follow‐up. GCS, recurrence of ICH, WML grade and cerebral atrophy were identified as independent prognostic factors of long‐term outcome.

## Graphical abstract

The long‐term functional outcome of cerebral amyloid angiopathy‐related hemorrhage (CAAH) patients is unclear. In our study, we enrolled consecutive CAAH patients from 2014 to 2020. Among the 141 CAAH patients, 76 (53.9%) achieved favorable outcomes and 19.9% of them died at 1‐year follow‐up. For the longer‐term follow‐up with a median observation time of 19.0 (interquartile range, 12.0–26.5) months, 71 (50.4%) patients obtained favorable outcomes and 33 (23.4%) of them died. Multivariable logistic regression analysis was performed to identify the prognostic factors associated with long‐term outcome. GCS, recurrence of ICH, WML grade 3‐4 and severe cerebral atrophy were identified as independent prognostic factors of long‐term outcome.

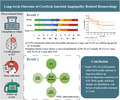

## 1 INTRODUCTION

Cerebral amyloid angiopathy (CAA) is a cerebral small vessel disease (SVD) and it is the most common cause of spontaneous lobar intracranial hemorrhage.^1^ Although cerebral amyloid angiopathy‐related hemorrhage (CAAH) patients have mild symptoms, a higher risk of recurrent and/or multiple hemorrhages may result in higher morbidity and mortality.^2^, ^3^, ^4^ Unfortunately, there are no effective treatment and prevention methods, and the surgical treatment during the acute phase is controversial.^5^, ^6^, ^7^ Therefore, understanding the outcomes of the disease and the prognostic factors first will help clinical decisions.

Up to now, most studies focus on the spontaneous intracranial hemorrhage (ICH) caused by various etiologies, but analysis caused by CAA is lacking. The few investigations on CAAH are retrospect studies with a small sample size or focus on the prognostics of the patients treated with surgery, which lack the data of the conservative treatment.^8^, ^9^, ^10^ Meanwhile, these studies pay more attention to the early and medium‐term prognosis of CAAH <1 year. Additionally, most of these studies are from the western countries.^11^, ^12^ Therefore, more evidences on long‐term functional outcomes of CAAH in China are needed.

For the prognostic factors of long‐term outcome in CAAH patients, previous studies were focused on demographics, medical history and the clinical manifestations.^8^, ^9^, ^10^ The influence of pre‐existing lesions in the brain were ignored. Recently, more and more studies demonstrated a stronger association between ICH and underlying cerebral SVD. The severity of neuroimaging abnormalities assessed by CT and MRI scans, such as cerebral white matter lesions (WML), lacunes, and brain atrophy, indicating the frailty of the underlying brain, which could influence susceptibility to and recovery from ICH.^2^, ^13^, ^14^ CAA, as a kind of SVD, characteristic neuroimaging biomarkers are also seen in the brain.^1^ However, it remains unclear how the imaging burdens are related to the outcome of CAAH in the long‐term follow‐up.

Hence, in this study, we retrospectively collected medical records of CAAH patients who received conservative treatment or/and surgical treatment. We aim to assess the long‐term functional outcomes among CAAH patients in China. Meanwhile, the prognostic factors associated with unfavorable outcomes were also investigated.

## 2 MATERIAL AND METHODS

### 2.1 Participants and ethics statements

This observational study was based on a prospective registry in Xuanwu Hospital, Capital Medical University, and Beijing Fengtai You ‘An men Hospital. The design of the study was approved by the Research Ethics Committees in both hospitals for the collecting of information. Informed consent was obtained from patients or legal representatives. Data were collected blinded to the results.

### 2.2 Procedures and data collection

We identified all consecutive patients with CAA‐related ICH from September 2014 to October 2020. All the patients received the treatments under the guidelines for the management of spontaneous ICH in China. In general, the patients received the general monitoring, the medicine of intracranial pressure‐reducing and/or BP‐lowering, treatment of complications, and physical exercise, according to the symptoms and signs. For the patients requiring surgery, neurosurgical hematoma evacuation or minimally invasive aspiration was performed. For the diagnosis of CAAH, we made the diagnosis by Boston criteria.^15^

Patients meeting the following criteria were included for statistical analysis. The inclusion criteria were (1) at least 55 years old, (2) all patients were probably or possibly CAA which was accessed by modified Boston criteria, (3) patients were diagnosed of cortical, or subcortical hemorrhage which were detected by non‐contrast computed tomography (CT) within 7 days from the presumed symptom onset. Patients were excluded if they met the criteria given below: (1) modified Rankin Scale (mRS) > 3 before symptom onset; (2) patients lost to follow‐up; (3) diagnosed with malignant tumor before admission and survival time of <1 year.

Patients' medical records were collected. In‐hospital details including clinical features and diagnosis were obtained through medical records. Follow‐up details were through telephone interviews or medical records in hospital at 3 months and 1 year. All the data were collected by the independent investigators blinded to the baseline performance of the patients and the details of the procedure.

For the medical history, hypertension, diabetes mellitus, hyperlipidemia, stroke, atrial fibrillation, heart disease, intracranial hemorrhage (ICH), ischemic stroke, current smoking, anticoagulation medicine, antiplatelet medicine and antihypertension medicine were recorded with self‐reported or diagnosed in hospital before ICH onset. Clinical status included Glasgow Coma Scale (GCS) [with a lower score indicating a worse conscious status], National Institutes of Health Stroke Scale (NIHSS) [from 0 to 42, with higher scores indicating more severe neurological deficits], blood pressure on admission, platelet and urea results.

Non‐contrast head CT scans were performed on arrival according to standardized techniques (recommended slice thickness, 5 mm). All imaging data were assessed by two experienced radiologists who were blinded to clinical outcome and other patient clinical information. Hematoma volume was determined by the formula of ellipsoids (A×B×C/2). White matter lesion (WML) grade was assigned using the scale described by Van Swieten et al. [from 0 to 4, with higher scores indicating more severe white matter lesion]. In grade 1 the abnormality was restricted to the region adjoining the ventricles; in grade 2, the increased hypodensity involved the entire region from the lateral ventricle to the cortex. Two regions: (1) around the anterior horns of the lateral ventricles; (2) around the posterior part of the cella media and the centrum semiovale) were scored separately and added together to give an overall value between 0 and 4 in statistical analysis.^16^ Lacunes were defined as subcortical rounded or ovoid, fluid‐filled (with cerebrospinal fluid signal intensity) lesions of 3–15 mm in diameter, consistent with a previous cerebral infarct or hemorrhage.^17^ For cerebral atrophy, four parameters, including visual inspection and linear measurements, were used on the contralateral side of the hematoma. Two linear measurements comprised the fontal ratio, and the third ventricle Sylvian fissure distance, which were described as before.^18^, ^19^ A higher frontal ratio and a shorter third ventricle Sylvian fissure distance indicated more advanced brain atrophy. Visual inspections included central and cortical atrophy which are both 3‐point scale (none, modest and severe).^20^

### 2.3 Outcome assessment

Patients’ outcomes were measured at 3‐month, 1‐year and beyond for longer‐term outcome determination. The functional outcomes were assessed by mRS. Favorable outcome was defined as the score of 0–3 at 1 year, while the unfavorable outcome was the score of 4–6. Mortality and recurrence of ICH were also evaluated during the follow‐up. Recurrence of ICH was recorded as self‐reported, or was diagnosed in the hospital.

### 2.4 Statistical analysis

All data were tested for normality using the Kolmogorov–Smirnov test. Normally distributed continuous variables are presented by the mean and standard deviation (SD), while the non‐normally distributed continuous variables were presented by median and interquartile range (IQR). The categorical variables are showed in percentages. Baseline variables on demographical and clinical data were compared by student's *t*‐test or Wilcoxon‐W‐test for continuous variables and Chi‐square test were used for categorical variables. Kaplan–Meier survival analysis was used to reflect the long‐term survival probability of CAAH patients. For the prognostic factors analysis, the logistic regression analysis was used to assess any independence factors. All statistics analysis was done by SPSS 21.0. Value of *p* < 0.05 was considered statistically significant.

## 3 RESULTS

**FIGURE 1:**
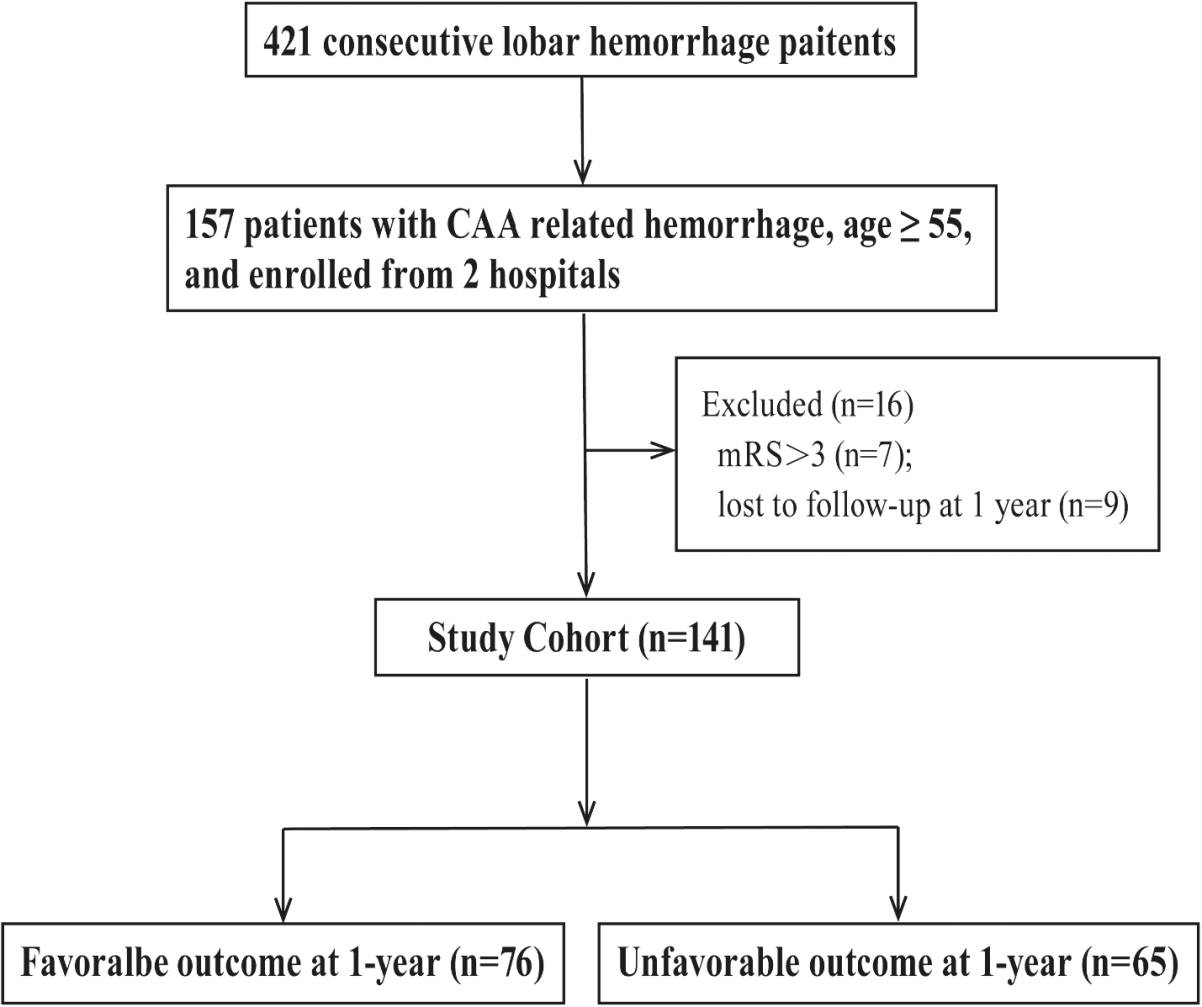
Flow diagram. This figure shows the enrollment information of patients in the present study, including the reasons for exclusion

A total of 157 consecutive CAAH patients were admitted to our institutions between January 2014 and October 2020. Sixteen patients did not fulfill the predefined criteria and were excluded, and thus 141 patients were enrolled and underwent primary analysis. Enrolled information is shown in Figure 1.

### 3.1 Baseline characteristics

Of the 141 enrolled patients, the median age was 71 (IQR 64.5–79.0) and 76 (53.9%) patients were male. The NIHSS on admission was 4.0 (IQR 1.0–15.0). The demographic and clinical characteristics of each group are shown in Table 1.

**TABLE 1:** Baseline characteristics in the study cohort

	Overall	Favorable	Unfavorable	*p* Value
	*n* = 141	*n* = 76	*n* = 65	
Demographics				
Age	71 (64.5–79.0)	67.5 (63.0–77.8)	76.0 (69.5–82.0)	<0.001
Male	76 (53.9)	43 (56.6)	33 (50.8)	0.490
Medical history				
Hypertension	57 (40.4)	51 (67.1)	33 (50.8)	0.049
Diabetes mellitus	27 (19.1)	13 (17.1)	14 (21.5)	0.505
Hyperlipidemia	19 (13.5)	15 (19.7)	4 (6.2)	0.019
Atrial fibrillation	5 (3.5)	2 (2.6)	3 (4.6)	0.525
HD	21 (14.9)	12 (15.8)	9 (13.8)	0.747
ICH	18 (12.8)	8 (10.5)	10 (15.4)	0.389
Ischemic stroke	23 (16.3)	12 (15.8)	11 (16.9)	0.856
Smoking	29 (20.6)	19 (25.0)	10 (15.4)	0.159
Anticoagulations medicine	4 (2.8)	2 (2.6)	2 (3.1)	0.874
Antiplatelet medicine	27 (19.1)	15 (19.7)	12 (18.5)	0.848
Antihypertension medicine	54 (38.3)	33 (43.4)	21 (32.3)	0.176
Clinical status				
GCS on admission	14.0 (11.0–15.0)	15.0 (14.0–15.0)	11.0 (8.0–13.5)	<0.001
NIHSS on admission	4.0 (1.0–15.0)	2.0 (1.0–4.0)	15.0 (4.5–24.5)	<0.001
Systolic BP, mmHg	153.9 ± 23.0	153.6 ± 26.1	154.2 ± 19.0	0.894
Diastolic BP, mmHg	85.0 (76.0–98.0)	84.0 (75.0–98.0)	85.0 (77.5–97.5)	0.433
Platelet, 10^9^/L	184.0 (149.0–224.0)	196.5 (163.0–244.8)	165.0 (130.0–199.0)	0.002
Urea，mmol/L	4.5 (3.6–5.9)	4.1 (3.3–5.5)	5.0 (3.9–6.3)	0.016
Hematoma volume, mL	28.3 (13.7–52.2)	18.9 (11.5–32.9)	49.9 (23.0–69.8)	<0.001
Midline shift, mm	0.0 (0.0–2.9)	0.0 (0.0–0.0)	0.4 (0.0–4.6)	0.009
Operation	41 (29.3)	14 (18.7)	27 (41.5)	0.003
Shape				0.001
Round	56 (40.9)	38 (52.8)	18 (27.7)	–
Lobular	36 (26.3)	10 (13.9)	26 (40.0)	–
Irregular	44 (32.1)	24 (33.3)	20 (30.8)	–
Hematoma location				
Frontal lobe	65 (47.1)	32 (43.2)	33 (51.6)	0.329
Parietal lobe	60 (43.5)	30 (40.5)	30 (46.9)	0.454
Temporal lobe	67 (48.6)	27 (36.5)	40 (62.5)	0.002
Occipital lobe	41 (29.7)	21 (28.4)	20 (31.3)	0.713
IVE	35 (24.8)	8 (10.5)	27 (41.5)	<0.001
SAH	44 (31.2)	15 (19.7)	29 (44.6)	0.001
Subdural hemorrhage	48 (34.0)	17 (22.4)	31 (47.7)	0.002
ICH Score	1.0 (0.0–2.0)	0.0 (0.0–1.0)	2.0 (2.0–3.0)	<0.001

*Note*: Data are *n* (%), mean ± SD or median (interquartile range).

Abbreviations: BP, blood pressure; GCS, Glasgow Coma Scale; HD, heart disease; ICH, intracranial hemorrhage; IVE, Intraventricular extension; NIHSS, National Institute of Health stroke scale; SAH, subarachnoid hemorrhage.

The patients in the unfavorable group showed a higher NIHSS and lower GCS scores on admission (*p* < 0.001 each). For the cerebral hematoma, the volume is larger (18.9 vs. 49.9 ml, *p* < 0.001) and more patients have intraventricular extension (*p* < 0.001) in unfavorable group.

### 3.2 Cerebral small vascular burden

SVD burdens based on CT scan are shown in Table 2. Compared with the unfavorable group, patients in the favorable group have a lower WML grade (*p* = 0.008) and more severe in all four measurements of atrophy (*p* < 0.05). There is no significant difference in the frequency of lacunes (*p* > 0.05).

**TABLE 2:** CT‐based cerebral small‐vessel disease biomarkers in the study cohort

	Overall	Favorable	Unfavorable	*p* Value
	*n* = 141	*n* = 76	*n* = 65	
WML grade				0.008
Grade 0	60 (44.1)	37 (50.7)	23 (36.5)	–
Grade 1	21 (15.4)	15 (20.5)	6 (9.5)	–
Grade 2	19 (14.0)	10 (13.7)	9 (14.3)	–
Grade 3 and 4	36 (26.5)	11 (15.1)	25 (39.7)	–
Lacunes				
With	83 (60.1)	48 (64.0)	35 (55.6)	0.313
>5	66 (47.8)	37 (49.3)	29 (46.0)	0.699
Atrophy				
Cortical atrophy				0.001
0	56 (40.6)	39 (52.0)	17 (27.0)	–
1	51 (37.0)	27 (36.0)	24 (38.1)	–
2	31 (22.5)	9 (12.0)	22 (34.9)	–
Central atrophy				<0.001
0	78 (56.5)	55 (73.3)	23 (36.5)	–
1	36 (26.1)	18 (24.0)	18 (28.6)	–
2	24 (17.4)	2 (2.7)	22 (34.9)	–
Frontal ratio, %	33.3 (31.6–35.7)	32.6 (31.1–34.0)	35.0 (32.5–36.6)	0.002
Third ventricle Sylvian fissure distance, mm	39.7 (38.2–41.7)	40.5 (39.1–42.3)	38.7 (36.7–40.2)	<0.001

*Note*: Data are *n* (%) or median (interquartile range).

Abbreviation: WML, white matter lesion.

### 3.3 Clinical outcomes

Compared with the 3‐month follow‐up, lesser patients have favorable outcomes (57.4% vs. 53.9%) and more patients died (13.5% vs. 19.9%) in the 1‐year follow‐up. For the longer‐term follow‐up, with a median observation period of 19.0 (IQR 12.0–26.5) months, 71 (50.4%) had favorable outcomes and 33 (23.4%) patients died. Twelve (8.5%) patients had a recurrence of ICH at longer‐term follow‐up (Table 3).

**TABLE 3:** Clinical outcomes in the study cohort

	Overall	Favorable outcome	Unfavorable outcome
3 months follow‐up			
mRS	2.0 (1.0–5.0)	1.0 (0.0–2.0)	5.0 (4.0–6.0)
mRS 0–3	81 (57.4)	75 (98.7)	6 (9.2)
Mortality	19 (13.5)	0 (0)	19 (29.2)
1 year follow‐up			
mRS	2.0 (1.0–5.0)	1.0 (0.0–1.0)	5.0 (5.0–6.0)
mRS 0–3	76 (53.9)	76 (100)	0 (0)
Mortality	28 (19.9)	0 (0)	28 (43.1)
Recurrence ICH	8 (5.7)	2 (2.6)	6 (9.2)
Longer‐term follow‐up			
Follow‐up months	19.0 (12.0–26.5)	20.0 (13.0–27.75)	17.0 (12.0–25.5)
mRS	3.0 (1.0–5.0)	1.0 (0.0–1.0)	6.0 (5.0–6.0)
mRS 0–3	71 (50.4)	71 (93.4)	0 (0)
Mortality	33 (23.4)	0 (0)	33 (50.8)
Recurrent ICH	12 (8.5)	4 (5.3)	8 (12.3)

*Note*: Data are *n* (%) or median (interquartile range).

Abbreviations: ICH, intracranial hemorrhage; mRS, modified Rankin Scale.

For the changes of mRS, 28 (19.8%) displayed an improvement in functional outcome of at least one point, and most of the patients (67.4%) were unchanged from 3‐month to 1‐year follow‐up. Meanwhile, only 4 (2.8%) patients showed an improvement from 1‐year to longer‐term follow‐up. To investigate the contributing factors to such improvement from 3‐month to 1‐year, multivariate logistic regression is used. After the statistical analysis, GCS on admission (2.354 (IQR [1.295–4.281], *p* = 0.005) is found to be contributing factors to such improvement (Table S1).

**FIGURE 2:**
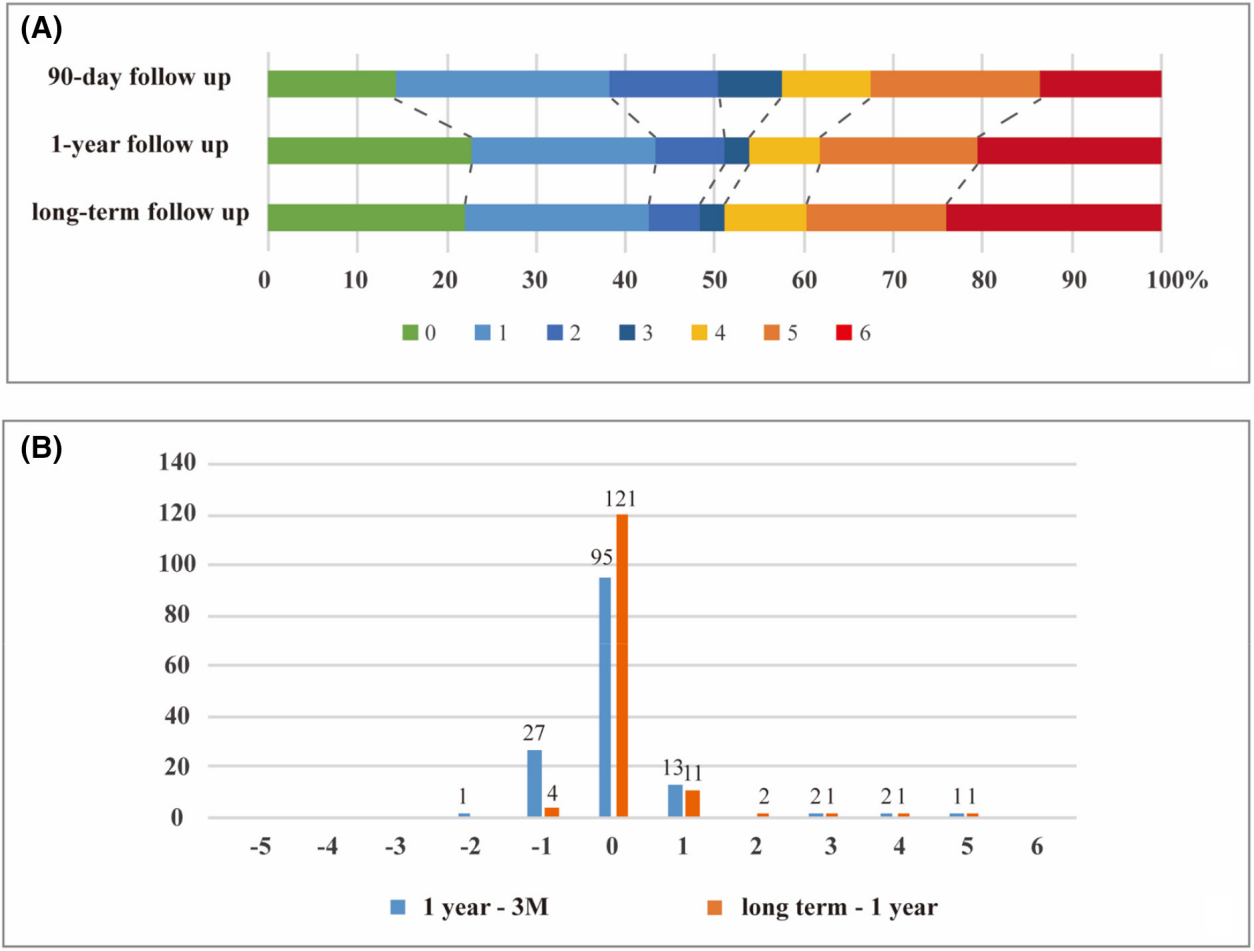
Clinical functional outcomes according to the modified Rankin Scale (mRS). (A) The raw distribution of score on mRS; (B) Changes of mRS during follow‐up. The abscissa represents the changes of mRS between two times. The ordinate represents the number of people

**FIGURE 3:**
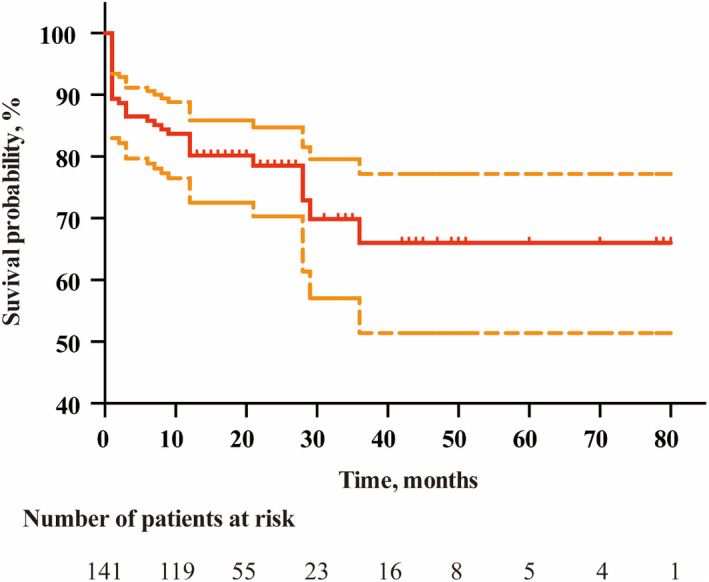
Kaplan–Meier curve for the long‐term survival probability of CAAH patients. The curve for long‐term follow‐up shows a survival probability of 86.5% at 3 months, 80.1% at 1‐year, and 78.5% at 2‐year after the symptoms onset. Censored events are represented by tick marks on this curve. The outer region represents the 95% confidence interval.

Detailed distributions of the mRS score are shown in Figure 2. Kaplan‐Merier Curve shows a survival probability of 86.5% at 3‐months, 80.1% at 1 year, and 78.5% at 2 years after ICH (Figure 3).

### 3.4 Prognostic factors

Firstly, the univariate logistic regression was performed to find variables that accounted for the unfavorable outcome (Table 4). Multivariate logistic regression analysis was used to adjust for confounding factors with *p* < 0.2. And GCS on admission (OR, 0.109; 95% CI, 0.021–0.556; *p* = 0.008), recurrence of ICH (OR, 2923.687; 95% CI, 6.282–1360730.14; *p* = 0.011), WML grade 3–4 (OR, 31.007; 95% CI, 1.041–923.573; *p* = 0.047), severe central atrophy (OR, 4220.303; 95% CI, 9.135–1949674.84; *p* = 0.008) were the independent factors of unfavorable outcomes at 1 year.

**TABLE 4:** Binary regression analysis regarding the unfavorable outcomes at 1‐year

	Univariate logistic regression		Multivariate logistic regression	
	Unadjusted OR (95% CI)	*p* Value	Adjusted OR (95% CI)	*p* Value
Age, per 1‐year increase	1.076 (1.033–1.122)	<0.001	0.904 (0.732–1.116)	0.349
Gender	1.264 (0.650–2.457)	0.491	–	–
Hypertension	0.506 (0.256–1.000)	0.050	–	–
Hyperlipidemia	0.267 (0.084–0.849)	0.025	0.016 (0–167.047)	0.381
GCS, per 1‐point increase	0.385 (0.275–0.541)	<0.001	0.109 (0.021–0.556)	0.008
NIHSS, per 1‐point increase	1.207 (1.125–1.296)	<0.001	1.112 (0.915–1.350)	0.286
SP, per 1 mmHg increase	1.001 (0.997–1.016)	0.895	–	–
Hemorrhage volume, per 1 ml increase	1.044 (1.026–1.063)	<0.001	1.015 (0.918–1.122)	0.776
IVE	6.039 (2.497–14.607)	<0.001	11.534 (0.499–266.628)	0.127
SAH	3.276 (1.552–6.915)	0.002	28.809 (0–1.160E+31)	0.923
Subdural	3.164 (1.530–6.544)	0.002	2.065 (0–7.944E+29)	0.983
Shape	0.710 (0.480–1.051)	0.087	0.742 (0.042–12.957)	0.838
Surgery	3.096 (1.445–6.633)	0.004	0.004 (0–1.725)	0.074
Recurrence of ICH	3.763 (0.732–19.329)	0.112	2923.687 (6.282–1360730.14)	0.011
WML grade, 1‐point increase	1.512 (1.144–1.998)	0.004	–	0.198
Grade 1	–	–	36.984 (0.538–2542.101)	0.094
Grade 2	–	–	17.246 (0.433–687.511)	0.130
Grade 3–4	–	–	31.007 (1.041–923.573)	0.047
Lacunes	0.703 (0.354–1.395)	0.314	1.216 (0.054–27.311)	0.902
Cortical atrophy, per 1‐point increase	2.325 (1.453–3.718)	<0.001	0.062 (0.004–1.047)	0.054
Central atrophy, 1‐point increase	3.846 (2.231–6.629)	<0.001	–	0.028
Mild	–	–	14.277 (0.553–368.812)	0.109
Severe	–	–	4220.303 (9.135–1949674.84)	0.008
Atrophy‐frontal ratio, per 1% increase	1.190 (1.057–1.341)	0.004	–	–
Atrophy third ventricle Sylvian fissure distance, per 1 mm increase	0.784 (0.687–0.895)	<0.001	0.515 (0.252–1.051)	0.068

Abbreviations: BP, blood pressure; GCS, Glasgow Coma Scale; ICH, intracranial hemorrhage; IVE, intraventricular extension; NIHSS, National Institute of Health Stroke Scale; SAH, subarachnoid hemorrhage; WML, white matter lesion.

For the longer‐term follow‐up, GCS on admission, subdural hemorrhage, and central atrophy were the independent factors. However, the SVD burdens were no longer the independent factors of mortality and recurrence of ICH at 1 year. Age and hemorrhage volume showed a significant relationship to mortality (Table S2).

## 4 DISCUSSION

In this observational study, we report the long‐term functional outcome of cerebral amyloid angiopathy‐related hemorrhage in China. After a long‐term follow‐up, 53.9% of patients achieve a favorable outcome at 1 year, and 50.4% at a median follow‐up period of 19 months. A total of 80.1% of patients survive at 1 year. Furthermore, we found that GCS on admission, recurrence of ICH, WML level 3‐4, and severe cerebral atrophy are the independent prognostic factors of functional outcome at 1‐year follow‐up among CAAH patients.

To our knowledge, this is the second published cohort of patients with CAAH about long‐term outcomes. The first study, released by Kaiser et al., focused on the percentage, risk factors, and comorbidities of patients suffering from CAAH.^10^ However, the number of patients in this study was smaller (74 patients) and the study did not reveal any information about SVD biomarkers based on CT. A Chinese study, included 367 CAAH patients treated with surgery. However, the patients younger than 55 years were included, as well as more patients with deep ICH (135/367 patients 36.7%) which did not meet the Boston criteria.^9^

Compared with western countries, the higher favorable outcomes, survival probability, and lower mortality are shown in our study. It may be caused by the several reasons below.^8^, ^10^ Firstly, there were significant differences between the studies' score data, including different patients' inclusion criteria, scoring systems, and definitions of favorable outcomes. Secondly, the patients seem to have a lower rate of hypertension and diabetes mellitus on admission, and mild symptoms compared with the patients in the West.^9^, ^10^, ^12^ Meanwhile, this phenomenon may relate to genetic or environmental exposures in different geographical regions, which may influence the prevalence, severity, and outcome of CAA.^11^

Interestingly, the results from the Kaplan Meier curve and the changes of mRS indicate the bulk of functional outcome improvement in CAAH patients at 3 months, and level off thereafter. In other words, long‐term survival for CAAH depends mainly on survival at 3 months. Previous studies have demonstrated that CAAH patients appear to have a better clinical outcome and recover more rapidly in the acute phase compared with other types of ICH.^2^, ^21^ It may be caused by the reasons given below. Firstly, compared with the patients with deep hematoma, patients with lobar hemorrhage seem to have more volume to offset the mass effect. Therefore, the hematoma volume has little effect on the prognostics unless there is a large amount of bleeding. Secondly, the neurological function of preclinical CAA affection appears to shift to neighboring brain structures before a lobar hemorrhage, resulting in a less‐than‐expected loss of neuro‐functions and more rapid immediate recovery.^9^, ^22^

When predicting the outcomes of our patients, GCS at admission but not the hematoma volume was the independent predictor of unfavorable outcomes at 1 year. GCS is an indicator of the level of consciousness of patients, and it can also reflect the severity of the disease. In our study, lower GCS indicates a poor prognosis at 1 year, and this result is consistent with the previous studies.^5^, ^10^ To be different from previous studies, hematoma volume in our study does not appear to be an independent risk factor for long‐term clinical outcome.^23^, ^24^, ^25^ This may be related to the following reasons. Firstly, the location of hematoma plays a pivotal role in prognosis as well. Previous studies showed that patients with lobar ICH are associated with improved neurologic outcomes compared with deep ICH.^26^, ^27^ Especially for the CAA patients in our study, most of them have varying degrees of brain atrophy which may further decrease the risk of brain herniation due to mass effect. Therefore, the hematoma volume has little effect on the prognostics unless there is a large amount of bleeding. Secondly, the brain injury after ICH because of the mass effect of hematoma and the associated edema and further injury due to the cellular toxicity seem to require a substantial time to resolve.^28^, ^29^ CAA, a small vascular disease, pre‐existing injury of the brain parenchyma and vessels may influence ICH recoveries, such as the impairment of white matter connectivity which affects learning, cognitive function, and rehabilitation.^14^, ^30^ Thus, the SVD markers may be a more important determinant of long‐term outcome.

In our study, we identify that WML grades 3–4 and brain atrophy are the prognostic factors of the unfavorable outcome at 1 year after ictus. In CAA, vascular amyloid damages the integrity of vascular and blood–brain barriers and then leads to microaneurysms or fibrinoid necrosis of the vascular wall. Long‐term ischemia and hypoxia in brain tissue lead to demyelination of white matter and brain atrophy which impacts connectivity and plasticity of the brain.^23^, ^31^, ^32^ Thus, the disruption of key brain networks is likely to impact the rehabilitation, learning, and cognitive reserve, which are important in the functional recovery after ICH. Meanwhile, WML and atrophy are likely to be associated with vascular injury severity and vascular risk factors which may impact the recurrence of stroke and other adverse vascular events. Interestingly, only WML grades 3–4 were the independent risk factors, which is in line with previous studies.^8^, ^9^, ^13^ This result indicates that the diffused pattern of WML but not the localized pattern may influence the clinical outcome at 1 year. Based on the animal model of CAA, a diffused pattern is shown in the late stage of the disease related to CMB, larger lobar hemorrhages, or focal ischemic insults.^33^ Thus, only the extensive WML may impact the poor outcome. Although there are some other evaluation methods of WML, such as the Fazekas Scale, ARWMC Scale, and Ylikoski Scale, which have more details on the image segmentation of lesions, MRI images are requested. Further study is needed. Besides, for the longer‐term follow‐up, severe atrophy instead of WML grade is the independent risk factor in the longer‐term follow‐up. It may be caused by the reasons given below. Firstly, although WML assessed at baseline primarily reflects the severity of the pre‐existing lesion, brain injury after intracerebral hemorrhage may also affect WML.^34^, ^35^ Meanwhile, for the median follow‐up period of 19 months, the severity of CAA seems to have a greater impact on the patients' prognosis. Therefore, WML may have a greater impact on the early or mid‐term recovery from ICH. Secondly, brain atrophy is a key mediator of cognitive impairment and functional outcomes. This kind of atrophy seems to be a combination of cortical gray matter and white matter, more reflective of the severity of CAA than the WML.^36^, ^37^ Therefore, brain atrophy, but not WML, is the independent risk factor in the longer‐term outcome.

There are some limitations to our study. Firstly, this is a retrospective study in two centers, and the relatively small sample size and evaluation index may not be able to represent all the CAAH patients in China. Secondly, recent studies suggested that iron dyshomeostasis following initial ICH injury is the reason for the neurological deficiency. Hence, perihematomal iron concentration measured by MRI is an independent predictor for ICH.^38^ Other MRI lesions in the remote brain regions (such as remote diffusion‐weighted imaging lesions, and contrast leakage distant from the hematoma) are also shown to influence the clinical outcomes in CAA patients.^37^, ^39^ However, due to the restriction of CT scans, we did not analyze these details in our study. In addition, some other SVD markers such as enlarged perivascular spaces, cerebral microbleeds, and cortical superficial siderosis were excluded from our analysis and restricted to CT scans. The effect of these markers requires future study. Overall, considering the absence of a clear conclusion on the clinical outcomes of CAAH patients, this study improves our understanding of the long‐term functional outcome and could at least provide some clinical evidence to guide the daily clinical practice.

In conclusion, over 50% of CAAH patients achieve favorable outcomes at long‐term follow‐up. GCS on admission, recurrence of ICH, and cerebral small vessel disease based on CT (WML level and cerebral atrophy) are identified as independent prognostic factors of long‐term outcomes.

## AUTHOR CONTRIBUTIONS

RC, MZ, XJ conceptualized and designed the study. HS collected the data. WH and JM analyzed the imaging data. RC analyzed the data. RC drafted the manuscript. MZ and XJ revised the manuscript.

## CONFLICT OF INTEREST

The author(s) declared no potential conflicts of interest with respect to the research, authorship, and/or publication of this article.

## Supporting information


Table S1‐S2

## Data Availability

All supporting data for this analysis are available from the corresponding author upon reasonable request.
