# Supplementary material for: Long‐term outcome of cerebral amyloid angiopathy‐related hemorrhage
Source: CNS Neurosci Ther. 2022 Aug 16;28(11):1829–37. doi: 10.1111/cns.13922 (PMC9532921; doi:10.1111/cns.13922)
Supplement: Supplementary file 1 — Table S1‐S2 [file CNS-28-1829-s001.docx]

**Supplemental material**

Supplemental Table 1. Multivariate logistic regression regarding improvement from 3-month to 1-year.

|  | Multivariate logistic regression | |
| --- | --- | --- |
|  | Adjusted OR (95% CI) | *P* value |
| Age, per 1-year increase | 1.045 (0.966-1.131) | 0.269 |
| Hypertension | 1.396 (0.396-4.920) | 0.603 |
| Diabetes Millitus | 0.735 (0.157-3.445) | 0.696 |
| Hyperlipidemia | 0.426 (0.064-2.823) | 0.377 |
| GCS, per 1-point increase | 2.354 (1.295-4.281) | 0.005 |
| NIHSS, per 1-point increase | 0.985 (0.887-1.093) | 0.773 |
| Hemorrhage volume, per 1 ml increase | 1.041 (0.999-1.085) | 0.056 |
| IVE | 1.714 (0.244-12.025) | 0.588 |
| SAH | 0.330 (0.012-8.755) | 0.507 |
| Subdural | 0.861 (0.044-16.872) | 0.921 |
| Surgery | 4.917 (0.845-28.600) | 0.076 |
| Recurrence of ICH within 1-year | - | 0.997 |
| WML grade, per 1-point increase | 1.205 (0.697-2.083) | 0.503 |
| Lacunes | 0.836 (0.228-3.068) | 0.787 |
| Cortical atrophy, per 1-point increase | 1.526 (0.549-4.240) | 0.417 |
| Central atrophy, per 1-point increase | 0.372 (0.119-1.158) | 0.088 |

GCS, Glasgow Coma Scale; NIHSS, National Institute of Health stroke scale; ICH, intracranial hemorrhage; IVE, Intraventricular extension; SAH, subarachnoid hemorrhage; WML, white matter lesion.

Supplemental Table 2. Multivariable regression analysis regarding the clinical outcomes after symptoms onset.

|  | Longer-term unfavorable outcome | | Recurrence of ICH | | Mortality | |
| --- | --- | --- | --- | --- | --- | --- |
|  | Unadjusted OR (95% CI) | *P* value | Adjusted OR (95% CI) | *P* value | Adjusted OR (95% CI) | *P* value |
| Age, per 1- year increase | 0.962 (0.864-1.071) | 0.477 | 0.984 (0.862-1.123) | 0.811 | 1.035 (0.957-1.120) | 0.386 |
| Hyperlipidemia | 0.094 (0.001-8.938) | 0.308 | -- | 0.999 | 0.077 (0.001-9.493) | 0.297 |
| GCS | 0.301 (0.127-0.711) | 0.006 | 0.891 (0.606-1.311) | 0.559 | 0.805 (0.609-1.065) | 0.129 |
| NIHSS, per 1-point increase | 1.110 (0.961-1.283) | 0.157 | 1.033 (0.936-1.140) | 0.520 | 0.993 (0.919-1.074) | 0.866 |
| Volume, per 1mL increase | 1.015 (0.959-1.075) | 0.599 | 0.982 (0.935-1.031) | 0.465 | 1.013 (0.984-1.042) | 0.390 |
| IVE | 5.127 (0.473-55.568) | 0.179 | 0.466 (0.038-5.765) | 0.552 | 0.498 (0.121-2.055) | 0.335 |
| SAH | 0.027 (0.001-1.413) | 0.074 | -- | 0.999 | 1.438 (0.078-26.525) | 0.807 |
| Subdural | 156.520(3.013-8130.659) | 0.012 | -- | 0.999 | 1.284 (0.078-21.089) | 0.861 |
| surgery | 0.047 (0.002-1.008) | 0.051 | 3.013 (0.289-31.430) | 0.356 | 0.478 (0.114-2.011) | 0.314 |
| WML level | 1.964 (0.943-5.750) | 0.071 | 1.820 (0.185-17.888) | 0.589 | 1.173 (0.678-2.032) | 0.568 |
| lacunes | 0.688 (0.082-1.398) | 0.730 | 1.820 (0.185-17.888) | 0.607 | 0.511 (0.118-2.211) | 0.369 |
| Cortical atrophy | 0.307 (0.067-25.924) | 0.127 | 3.271 (0.786-13.612) | 0.103 | 0.572 (0.226-1.449) | 0.239 |
| Central atrophy | - | 0.021 | 0.548 (0.143-2.097) | 0.380 | 1.937 (0.762-4.922) | 0.165 |
| Mild | 3.019 (0.352-1706.478) | 0.314 | - | - | - | - |
| Severe | 77.324 (3.504-1706.478) | 0.006 | - | - | - | - |
| Atrophy- third ventricle Sylvian fissure distance, per 1mm increase | 0.710 (0.504-1.000) | 0.050 | - |  | - | - |
| ICH recurrence | 49.311 (2.430-1000.487) | 0.011 | - | - | 13.874 (3.925-49.046) | < 0.001 |

GCS, Glasgow Coma Scale; NIHSS, National Institute of Health stroke scale; ICH, intracranial hemorrhage; BP, blood pressure; IVE, Intraventricular extension; SAH, subarachnoid hemorrhage; WML, white matter lesion.
